# URMC Universal Depression Screening Initiative: Patient Reported Outcome Assessments to Promote a Person-Centered Biopsychosocial Population Health Management Strategy

**DOI:** 10.3389/fpsyt.2021.796499

**Published:** 2022-01-11

**Authors:** Kimberly A. Van Orden, Julie Lutz, Kenneth R. Conner, Caroline Silva, Michael J. Hasselberg, Kathleen Fear, Allison W. Leadley, Marsha N. Wittink, Judith F. Baumhauer

**Affiliations:** ^1^Department of Psychiatry, University of Rochester Medical Center, Rochester, NY, United States; ^2^Department of Emergency Medicine, University of Rochester Medical Center, Rochester, NY, United States; ^3^Department of Orthopaedics and Rehabilitation, University of Rochester Medical Center, Rochester, NY, United States; ^4^Department of Pediatric Allergy and Immunology, University of Rochester Medical Center, Rochester, NY, United States

**Keywords:** depression, patient-reported outcomes measure (PRO), patient-centered care, biopsychosocial, health system, patient reported outcomes measurement information system (PROMIS), PHQ-9, depression screening

## Abstract

**Background:** Patient-reported outcomes (PROs) can promote person-centered biopsychosocial health care by measuring outcomes that matter to patients, including functioning and well-being. Data support feasibility and acceptability of PRO administration as part of routine clinical care, but less is known about its effects on population health, including detection of unmet healthcare needs. Our objectives were to examine differences in rates of clinically significant depression across sociodemographic groups and clinical settings from universal depression screens in a large health system, estimate the number of patients with untreated depression detected by screenings, and examine associations between biopsychosocial PROs—physical, psychological, and social health.

**Methods:** We analyzed data from over 200,000 adult patients who completed depression screens—either PROMIS (Patient Reported Outcomes Measurement Information System) or PHQ-2/9—as part of routine outpatient care.

**Results:** Depression screens were positive in 14.2% of the sample, with more positive screens among younger vs. older adults, women vs. men, non-White vs. White, and Hispanics vs. non-Hispanics. These same sociodemographic indicators, as well as completing screening in primary care (vs. specialty care) were also associated with greater likelihood of detected depression in the medical record.

**Discussion:** Universal screening for depression symptoms throughout a large health system appears acceptable and has the potential to detect depression in diverse patient populations outside of behavioral health. Expanded delivery of PROs to include physical and social health as well as depression should be explored to develop a clinically-relevant model for addressing patients' biopsychosocial needs in an integrated fashion across the health system.

## Introduction

Patient-reported outcomes (PROs) can promote person-centered biopsychosocial health care by measuring outcomes that matter to patients, including functioning and well-being (1). UR Medicine implemented PRO screenings for physical, social and mental health in a range of settings with integration into the electronic medical record (EMR) for clinicians to use during patient visits (2). Data support feasibility and acceptability of PRO administration as part of routine clinical care, with positive effects on patient experience (2, 3). Less is known about its effects on population health, including detection of unmet healthcare needs, especially for psychosocial health indicators.

The current study presents data from over 200,000 patients who completed depression PRO screens—either PROMIS (Patient Reported Outcomes Measurement Information System) (4) or PHQ-2/9 (5, 6)—as part of routine outpatient care. We chose to focus on depression screening in adults because depressive symptoms impact numerous health outcomes, contribute to significant disability, and depression is a recommended screening domain in primary care. Well-validated systems for tracking depression as a PRO may improve identification of depressive disorders outside behavioral health settings and provide information that can improve outcomes for numerous health conditions in a biopsychosocial model of care.

Both the PROMIS depression screen (7–11) and the PHQ-9 (12) have been validated across diverse sociodemographic groups and have been shown to reliably detect clinically significant depressive symptoms across gender, race/ethnicity, and age. In addition, although these studies using these measures reliably demonstrate higher rates of clinically depressive symptoms among women, differences across race/ethnicity are inconsistent across studies. Major Depressive Disorder is often reported as less prevalent among racial/ethnic minorities compared to non-Hispanic Whites in community-based samples when controlling for socioeconomic status; (13–15) however, some studies examining the PHQ-9 have demonstrated higher rates of clinically significant depressive symptoms among Black and Hispanic adults (12, 16, 17). Research is needed to compare rates of clinically significant depressive symptoms in clinical (vs. community) samples for patients of diverse racial and ethnic backgrounds. Little is known about how effective universal screening for depression may be in detecting untreated depression across diverse sociodemographic groups and clinical settings, especially with PROs. In addition, the PROMIS measures of patient-reported outcomes, developed as part of the NIH Roadmap initiative, include not only psychological dimensions of health (e.g., depression) but also physical and social domains as well. There could be advantages to examining biopsychosocial dimensions of health and functioning during brief PROs screens, but little is known about such a strategy.

Data from this study were collected as part of universal screening for depression conducted outside of behavioral health clinics using a web-based platform called UR VOICE (University of Rochester Validated Outcomes In the Clinical Experience), which runs on tablets provided to patients at check-in for office visits (2, 18, 19). UR VOICE administers the Patient Reported Outcomes Measurement Information System (PROMIS) measures as well as the PHQ-2/9. URMC uses PROs to measure biopsychosocial conditions across the health system, including depression and anxiety, physical function, social roles and activities, and pain interference. PRO data is available immediately to clinical providers and viewed in the electronic medical record and can be discussed during clinic visits with patients to promote shared decision making. Given these data were collected as part of routine standard of care, clinical settings selected PROs most relevant to their patient population, with variability in measures across settings.

For this study, we focused on a cohort of patients who completed depression screens *via* universal screening in outpatient clinics, either PROMIS Depression or PHQ-2/9. We used a validated crosswalk of PROMIS depression and PHQ-2/9 scores (20, 21) to analyze depression screens with a standardized T-score metric and associated norms. We defined positive depression screens as T scores of 60 or greater, which corresponds to a symptom severity level of moderate (or severe). This was selected as the threshold for clinical significance given that this level of symptomatology is reliably associated with the presence of a Major Depressive Episode (6, 21). We examined the proportion of patients with positive screens who did not have documented mood disorder diagnoses in the year prior to or following the screen as an indicator of whether the diagnosis was identified (or missed) by providers (and thus whether treatment may have been provided). Our objectives were:

1. To examine the number of patients with clinically significant depressive symptoms detected by universal screening with PROs in a large health system (including variation across sociodemographic groups and clinical settings);
2. To estimate the number of patients with clinically-significant depressive symptoms detected by the screening who were likely not receiving treatment across sociodemographic groups and clinical settings; and,
3. To examine associations between biopsychosocial PROs—physical, psychological (depression), and social health.

## Methods

### Participants

Data come from the EMRs of 206,468 adult patients (age 18 or older) in the UR Medicine system who completed depression screens (either PROMIS depression or PHQ-2/9) prior to healthcare visits from 2015 to 2018 as part of universal screening in several settings, including primary care, orthopedics, urology, and pain clinics. This study was approved by the University of Rochester IRB; specifically, IRB approval was granted for use of PRO data gathered as part of routine clinical care, with de-identified data provided to URMC researchers.

### Procedures

Data used in these analyses, including PRO scores for depression, physical function, and social function, were extracted from the EMR. The index visit was the visit for which the patient completed the first documented depression screen using UR VOICE at check-in. Some patients were established patients completing the tablet screening for the first time, while others were new patients who began care at the clinic when screening procedures were in place. Many patients completed follow-up depression screens, but those data are not used in the current study.

### Measures

Demographics were extracted for the index visit (age, sex, race, and ethnicity). Mood disorder diagnoses were extracted from the EMR with a positive diagnosis if any of the following ICD codes were present in the 12-months prior to and following the index visit date: any F30 codes (Manic episode); any F31 codes (Bipolar disorder); any F32 codes (Major depressive disorder, single episode); any F33 codes (Major depressive disorder, recurrent); any F34 codes [Persistent mood (affective) disorders]; and any F39 codes [Unspecified mood (affective) disorders].

#### Depression PRO Screens

Scores for depression were extracted from the EMR. Patient responses generate a standardized psychometric T score comparing the patient's responses to the population mean, with a T score of 50 corresponding to the mean of the reference population and a standard deviation of 10. All patients completed depression screens at the index visit. Patients who completed the depression screen in primary care clinics completed the Patient Health Questionnaire-2/9 (the tablet stopped administration after PHQ-2 if patients did not screen positive on those items), while patients in all other clinical settings completed the PROMIS depression computerized adaptive test (CAT). PHQ-2/9 scores were converted to T scores using validated “cross-walk” tables (21). Depression T scores ≥ 60 (one standard deviation above average, considered “moderate” depression) were coded as positive screens. For patients who completed the PHQ-9, this corresponds to a score of at least 10.

#### Physical and Social Function PRO Screens

Patients in some clinics also completed additional PROMIS measures. Patients who completed depression screens in orthopedic clinics also completed the PROMIS Physical Function CAT. Patients who completed depression screens in urology clinics also completed the PROMIS “Social Roles and Activities” CAT, which assesses social functioning (part of the PROMIS “Social Heath” domain). As with depression PROs, patient responses generate T scores with a mean of 50 and standard deviation of 10, but for these domains, higher scores indicate greater (better) physical and social function.

### Data Analysis

For our first objective, to examine rates of clinically significant depression (using PROs) across sociodemographic groups and clinical settings, we used *t*-tests and analyses of variance (ANOVA) to compare Depression T scores across demographic groups and clinical settings; we also conducted comparable analyses using a binary variable representing the presence of absence of a positive depression screen and conducted chi-square tests to compare the proportion of positive screens across sociodemographic groups and clinical settings where screens were conducted. For our second objective focused on the number of patients with positive screens, we examined the proportion who had documented mood disorder diagnoses in order to estimate the number of “missed” diagnoses (i.e., proportion of all patients who screened positive for depression who did not have documented mood disorder diagnoses) across demographic groups and clinical settings; to do so, we used chi-square tests. We also examined differences in demographic make-up of patients who were screened across different clinical settings using chi-square tests, and then used analyses of covariance (ANCOVA) and logistic regression to further examine differences in screens and diagnoses across clinical setting while adjusting for demographic variables given significant differences in demographics across clinical settings (e.g., older age in orthopedic clinics). This allowed us to test whether clinic differences were primarily due to variations in population demographics by clinical setting. For our third objective, we computed correlation coefficients between the biopsychosocial PROs scores and also compared depression T-score means on physical and social health for those with and without positive depression screens.

## Results

Table 1 presents sample characteristics, including sex (55.9% female), race (81.4% White), ethnicity (91.5% non-Hispanic), and age (mean 51.29 years, *SD* = 17.85). UR Medicine is in the city of Rochester, NY, within Monroe County: the race and ethnicity distribution of our sample is less diverse than the city of Rochester, NY, which has a higher proportion of Black (39.8%) and Hispanic (19.2%) individuals, but comparable to that of Monroe County (16.2% Black, 9.2% Hispanic/Latino). Most screens were conducted in orthopedic clinics, as these clinics were the first to initiate standardized procedures for collecting PROs.

**Table 1:** Sample characteristics.

**Variable**	**M (SD) or *n* (%)**
**Sex**	
Female	115,332 (55.9%)
Male	91,129 (44.1%)
Missing/Other	7 (0.0%)
**Race**	
White	168,089 (81.4%)
Asian	2,871 (1.4%)
Black	25,314 (12.3%)
Other	7,333 (3.6%)
Missing/Unknown	2,861 (1.4%)
**Ethnicity**	
Not Hispanic	188,817 (91.5%)
Hispanic	8,813 (4.3%)
Missing/Unknown	8,838 (4.3%)
**Age in years**	51.29 (17.85)
**Age groups**	
Younger (<65 years)	154,730 (74.9%)
Older (≥65 years)	51,738 (25.1%)
**Clinics**	
Orthopedics	135,182 (65.5%)
Surgery	6,533 (3.2%)
Primary care	27,112 (13.1%)
Oncology	3,397 (1.6%)
Specialty clinics	26,390 (12.8%)
Missing/multiple/other	7,854 (3.8%)
**PROMIS depression T score**	48.87 (9.94)
**PROMIS depression screen**	
Positive (T ≥ 60)	29,314 (14.2%)
Negative (T < 60)	177,154 (85.8%)
**PROMIS physical function T score**, ***n*** **=** **179,094**	43.53 (10.23)
**PROMIS social satisfaction T score**, ***n*** **=** **1,425**	47.63 (10.55)
**Mood disorder diagnosis**	
Diagnosis	26,946 (13.1%)
No diagnosis	179,522 (86.9%)
**PROMIS depression/diagnosis match**	
Positive screen, diagnosed	9,335 (4.5%)
Positive screen, not diagnosed	19,979 (9.7%)
Negative screen, diagnosed	17,611 (8.5%)
Negative screen, not diagnosed	159,543 (77.3%)

*Sample N = 206,468. If variable had missing data for some subjects, completed n is noted. The following clinic groups included: Orthopedics/Pain (Orthopedic Surgery, Pain Medicine, Physical/Occupational Therapy, Physical Medicine and Rehabilitation, Podiatry, Orthotics/Prosthetics/Pedorthis, Anesthesiology), Surgery (Cardiothoracic Surgery, Colon and Rectal Surgery, General Surgery, Neurosurgery, Plastic Surgery, Vascular Surgery), Primary Care (Family Medicine, Geriatric Medicine, Internal Medicine), Oncology (Oncology, Pediatric Oncology, Radiation Oncology, Surgical Oncology), Specialty Clinics (Allergy/Immunology/Rheumatology, Cardiology, Dermatology, Endocrinology, Infectious Diseases, Nephrology, Neurology, Ophthalmology, Urology, Obstetrics and Gynecology, Transplant)*.

Results for our first objective indicated that depression screens were positive in 14.2% of the sample (*n* = 29,314 out of 206,468 patients). Significant differences were found in the prevalence of positive screens as a function of demographics (Table 2, all statistically significant, *p* < 0.001): more positive screens among younger vs. older adults (15.1 vs. 11.6%), and women vs. men (16.0 vs. 12.0%), consistent with prior research on sociodemographic differences in the prevalence of depression. Of note, there were significantly more positive screens among non-White vs. White (22.4 vs. 12.5%) and Hispanics vs. non-Hispanics (26.4 vs. 13.7%). Specifically, patients of “Other races” (25.9%) had significantly more positive screens than White, Black, or Asian patients; due to limitations with data on race in the EMR, it is not possible to characterize those listed as “Other,” as this may refer to patients who select “Other” *via* the patient portal, as well as clinic staff or providers selecting “Other” for the patient. This category may include bi-racial individuals, those who identity most with a specific nationality, as well as those who identify (or are identified as) primarily as Hispanic/Latino rather than a specific race. Positive screens were also significantly more prevalent among Black patients (22.5%) than White (13.0%) or Asian (12.5%) patients, who did not differ significantly from each other.

**Table 2:** Depression T scores and number of positive screens by demographic groups.

	**Depression T scores**			**Positive screens**		**Diagnoses**		**Missed diagnoses^b^**	
**Demographic group**	**M (SD)**	***t* (df) or *F* (df)**	**95% CI of mean difference^**a**^**	***n* (% within group)**	**χ^2^ (df)**	***n* (% within group)**	**χ^2^ (df)**	***n* (% within group)**	**χ^2^ (df)**
**Age group**		9.09 (206,466)^***^	0.36, 0.56		371.51 (1)^***^		204.82 (1)^***^		152.07 (1)^***^
Younger	48.99 (10.16)			23,293 (15.1%)		21,143 (13.7%)		15,478 (66.4%)	
Older	48.53 (9.22)			6,021 (11.6%)		5,803 (11.2%)		4,501 (74.8%)	
**Sex**		50.98 (206,459)^***^	2.15, 2.32		668.02 (1)^***^		2,289.10 (1)^***^		230.11 (1)^***^
Female	49.86 (9.84)			18,410 (16.0%)		18,687 (16.2%)		11,963 (65.0%)	
Male	47.63 (9.91)			10,903 (12.0%)		8,256 (9.1%)		8,016 (73.5%)	
**Race**		911.05 (3,203,603)^***^	–		2,657.68 (3)^***^		1,094.68 (3)^***^		91.37 (3)^***^
White	48.38 (9.69)			20,961 (12.5%)		20,396 (12.1%)		14,530 (69.3%)	
Black	51.31 (10.62)			5,685 (22.5%)		4,604 (18.2%)		3,666 (64.5%)	
Asian	48.08 (9.71)^†^			374 (13.0%)		231 (8.0%)		290 (77.5%)	
Other	52.05 (11.28)			1,900 (25.9%)		1,476 (20.1%)		1,187 (62.5%)	
**Race (binary)**					2,376.49 (1)^***^		816.82 (1)^***^		58.60 (1)^***^
White				20,961 (12.5%)		20,396 (12.1%)		14,530 (69.3%)	
Non-White				7,959 (22.4%)		6,311 (17.8%)		5,143 (64.6%)	
**Ethnicity**		32.09 (197,628)^***^	3.25, 3.68		1,121.57 (1)^***^		421.11 (1)^***^		35.53 (1)^***^
Not Hispanic	48.74 (9.84)			25,800 (14.2%)		24,326 (12.9%)		17,656 (68.4%)	
Hispanic	52.20 (11.30)			2,328 (26.4%)		1,803 (20.5%)		1,453 (62.4%)	

*For ANOVAs, group differences (determined using Bonferroni test) are indicated with superscripts (†); groups with same superscripts are not significantly different*.

a *Available for t-tests only*.

b *Sample for these analyses includes positive PROMIS Depression screens only. “Missed diagnosis” refers to those who screened positive but did not receive a mood disorder diagnosis*.

*** *p < 0.001*.

For our second objective, we examined the proportion of patients with positive screens who had documented mood disorder diagnoses within the year prior/following the visit at which PRO screens were conducted. Of the 29,314 patients with positive screens, 31.8% (*n* = 9,335) had documented mood disorder diagnoses, while 68.2% (*n* = 19,979) did not (see bottom of Table 1). Significant differences emerged regarding likelihood of documented mood disorder diagnoses among those with positive screens as a function of demographics (Table 2), with greater likelihood of diagnoses among younger vs. older adults (33.6 vs. 25.2%), female vs. male patients (35 vs. 26.5%), non-White vs. White (35.4 vs. 30.7%), and Hispanic vs. non-Hispanic patients (37.6 vs. 31.6%). Specifically, Asian patients (22.5%) were less likely to have a diagnosis (given a positive screen) than White (30.6%), Black (35.5%), or Other race (37.5%) patients. Other race and Black patients were significantly more likely to have a diagnosis than White patients as well, although did not differ from each other.

Significant differences also emerged regarding likelihood of documented mood disorder diagnoses among those with positive screens as a function of clinical setting where the screen was conducted (Table 3), with greater likelihood of diagnoses among those with positive screens in primary care clinics (67.8%) compared with specialty clinics (26.4%) and lower likelihood of diagnoses in orthopedics (23.3%) compared with all other clinics combined (46.8%). To test whether differences by clinical setting were due to differing demographics, we examined age, sex, race, and ethnicity by clinical setting, and found significant results for all comparisons (Supplementary Table 1), with the largest differences being greater diversity in race and ethnicity in primary care clinics and older age in oncology clinics. After accounting for these demographic variables (Table 4), oncology no longer significantly differed in diagnosis rate among those who screened positive compared to all other clinics. However, even when adjusting for demographic differences, patients screening positive in primary care were 83% *less* likely to have “missed diagnoses” (i.e., positive screens but no diagnoses) compared to specialty clinics, and patients screening positive in orthopedics were almost three times as likely to be missing diagnoses compared to all other clinics combined.

**Table 3:** Depression mean T scores and number of positive screens by clinic setting.

	**Depression T scores**			**Positive screens**		**Diagnoses**		**Missed diagnoses** ^**a**^	
**Clinic comparison**	**M (SD)**	***t* (df)**	**95% CI of mean difference**	***n* (% within group)**	**χ^2^ (df)**	***n* (% within group)**	**χ^2^ (df)**	***n* (% within group)**	**χ^2^ (df)**
Primary care vs. specialty		22.67 (53,500)^***^	1.70 to 2.02		188.04 (1)^***^		2,948.84 (1)^***^		1,337.04 (1)^***^
Primary care	50.37 (9.26)			4,605 (17.0%)		8,073 (29.8%)		1,482 (32.2%)	
Specialty	48.51 (9.67)			3,368 (12.8%)		2,861 (10.8%)		2,480 (73.6%)	
Orthopedics vs. others^b^		19.14 (198,612)^***^	0.82 to 1.00		66.54 (1)^***^		3,409.06 (1)^***^		1,604.10 (1)^***^
Orthopedics	48.52 (10.02)			18,287 (13.5%)		13,210 (9.8%)		14,033 (76.7%)	
Others	49.43 (9.58)			9,444 (14.9%)		12,147 (19.1%)		5,025 (53.2%)	
Oncology vs. others^b^		0.71 (198,612)	−0.21 to 0.46		11.63 (1)^**^		7.75 (1)^**^		5.12 (1)^*^
Oncology	48.69 (9.42)			406 (12.0%)		380 (11.2%)		300 (73.9%)	
Others	48.81 (9.90)			27,325 (14.0%)		24,977 (12.8%)		18,758 (68.7%)	

a *Calculated out of positive screens only. “Missed diagnosis” refers to those who screened positive but did not receive a mood disorder diagnosis*.

b *Others includes all other clinics besides the comparison*.

* *p < 0.05*.

** *p < 0.01*.

*** *p < 0.001*.

**Table 4:** Depression mean T scores and number of positive screens by clinic setting, adjusting for demographics.

	**Depression T scores** ^**b**^		**Positive screens**			**Diagnoses**			**Missed diagnoses** ^**c**^		
**Models**	***F* (df)**	**Partial η^2^**	**Step**	**Odds**	**95% CI**	**Step**	**Odds**	**95% CI**	**Step**	**Odds**	**95% CI**
			**χ^2^ (df)^**a**^**	**ratio**		**χ^2^ (df)^**a**^**	**ratio**		**χ^2^ (df)^**a**^**	**ratio**	
Primary care vs. specialty^d^	222.40 (149,925)	0.004	52.43 (1)	1.22	1.16–1.29	2,453.75 (1)^***^	3.45	3.27–3.63	1,166.11 (1)^***^	0.17	0.15–0.19
Orthopedics vs. others	76.90 (1,189,369)^***^	0.000	3.25 (1)	1.03	1.00–1.06	2,633.34 (1)^***^	0.48	0.46–0.49	1,457.96 (1)^***^	2.91	2.75–3.07
Oncology vs. others	13.60 (1,189,369)^***^	0.000	0.01 (1)	1.01	0.90–1.13	6.60 (1)^**^	1.16	1.04–1.30	0.001 (1)	1.00	0.79–1.26

*All models are controlling/covarying Age Group, Sex, Race, and Ethnicity*.

a *In logistic regressions, demographic covariates were entered in Step 1, and clinic comparison was entered in Step 2. Chi square statistics for the addition of Step 2 (i.e., addition of the clinic comparison) are reported*.

b *Estimated marginal mean T scores were higher in Primary Care (M = 50.15, SE = .06) vs. Specialty Clinics (M = 48.80, SE = 0.06), lower in Orthopedics (M = 48.59, SE = 0.02) vs. Others (M = 49.12, SE = 0.04), and higher in Oncology (M = 49.47, SE = 0.18) vs. Others (M = 48.82, SE = 0.02)*.

c *Calculated out of positive screens only. “Missed diagnosis” refers to those who screened positive but did not receive a mood disorder diagnosis*.

d *Comparison group is Specialty Clinics*.

** *p < 0.01*.

*** *p < 0.001*.

For our third objective, we examined the association between the three PRO domains. Depression scores were negatively associated with Physical and Social Health (*r* = −0.420, *r* = −0.542, *p* < 0.001). Individuals with positive screens had significantly lower Physical Function and Social Health than those with negative Depression screens (35.60 vs. 44.80 and 39.53 vs. 49.79, *p* < 0.001).

## Discussion

Universal screening for depression is becoming standard of care in many clinical settings. Our results support the utility of universal depression screening for detecting untreated depression across diverse sociodemographic groups and clinical settings. Our results are consistent with prior studies regarding differences in prevalence of clinically significant depressive symptoms across sociodemographic groups, with a greater number of positive depression screens among younger adults, women, non-White patients (particularly Black and Other race patients), and Hispanic adults. These same sociodemographic indicators are also associated with greater likelihood of a documented mood disorder diagnosis in the medical record around the time of screening for those with positive screens (i.e., whether depression may have been detected and treated).

Regarding racial/ethnic differences in scores on the PROMIS depression screen, our results are consistent with some studies using the PHQ-9 indicating greater depression severity among racial/ethnic minorities. It may be that depressive symptom severity is higher among racial/ethnic minorities presenting to medical settings than in community-based, nationally representative samples. At the same time, these differences may reflect findings from other studies that indicate that although prevalence of Major Depressive Disorder may be lower among racial/ethnic minorities, disease burden (e.g., longer chronicity of disease) may be greater for racial/minorities (15, 22). Our results also point to the need for more fine-grained (and patient-centered) assessments of race and ethnicity given our finding for high rates of positive screens among those of “Other races.” Overall, our results suggest an opportunity to reduce health disparities in depression treatment given that a greater number of patients without documented mood disorders were detected by universal screening in groups with known health disparities in depression care.

Our results also indicate differences across clinical settings with regards to likelihood of detection of depression (and presumably treatment) among those with positive screens. After adjusting for demographic differences in clinical settings, patients screened in primary care were most likely to have received mood disorder diagnoses around the time of screening, indicating potential depression treatment within the healthcare system. These results could indicate that primary care clinicians were most likely to provide depression-focused care in response to universal screening or that depression had already been detected and treated for patients with positive screens in primary care. There are several reasons this might be the case. First, it is possible that patients in primary care were more likely to complete follow-up screens (with repeated positive screens), which we were unable to examine in this paper; this interpretation would be consistent with providers using a “watchful waiting” management approach (23). Second, it is possible that patients are more honest in rating their depression symptoms in a primary care setting where they may already have a trusted relationship with a physician who is trained to assess and attend to biopsychosocial needs, and/or has additional resources to do so (e.g., embedded mental health providers) (24). Third, patients seen in specialty clinics who do not have a primary care physician in the same health system have less complete data in the EMR: PCPs will often document a broader variety of health problems and concerns (than specialty providers) and also refer for specialty care within their healthcare network. Providers in specialty clinics may not document depression care if it is not seen as relevant to the patient's presenting problem; thus our data for specialty clinics may be less complete compared to primary care. Future research examining whether patients receive primary care in a different health system as well as research specifically examining provider behavior in response to depression screening, and how it varies by specialty, training and practice-level mental health resources, could address this issue. Visit recordings of clinician-patient communication around screening results (25) as well as patient feedback on provider communication (26) can be used to inform best practices and procedures for maximizing utility of universal depression screening tailored to the needs of clinical settings. Examining provider behavior in response to alerts provided in the EMR in response to elevated PRO scores could also provide clues as to how screens are responded to and also suggest strategies to address unmet biopsychosocial needs, such as health-system support for care managers to follow-up on PRO scores and digital health programming for depression self-management.

Our results regarding sociodemographic differences may indicate disparities in diagnosis (and potentially treatment) among patients with significant symptoms (i.e., under-detection) but could also indicate differences in the accuracy of the PROMIS measure in detecting clinically significant symptoms across diverse groups. For example, positive screens were less common among older (vs. younger) adults and, given a positive screen, older adults were less likely to receive a depression diagnosis. Given differing symptom profiles for depression in older adults, it is possible that age-specific norms could more accurately identify clinically-relevant depressive symptoms in this population by using older adults as the reference population. The utility of sex and race/ethnicity-specific norms could also be explored. Future work is needed to better understand the nature of discordant screens/diagnoses (i.e., positive screen/absent diagnosis and negative screen/positive diagnosis), including confirming whether treatment was considered but not provided because it was not needed or not desired by patients; whether further assessment was conducted to evaluate the need for treatment; and what specific treatment options were considered, provided, and received (e.g., antidepressant medications, referral to psychiatry clinics). Given associations of depression scores with physical and social health, future work is also needed to examine whether administering brief biopsychosocial PRO assessments with physical, psychological, and social domains might most accurately identify patients with untreated depression and other unmet health needs. For example, for some patients, depression may manifest with more physical or social health challenges than psychological or emotional symptoms (27, 28). Supplementing depression screens—that capture psychological health—with physical function and social health screens may have additional benefits beyond depression care, to address more fully a patient's biopsychosocial health profile that may impact numerous domains of function, symptoms, behaviors, and feelings.

Limitations of our study include that the demographic breakdown of our sample is representative of the settings in which PROs were rolled out, with unclear generalizability to the medical center more broadly. Future work is needed to study processes and results from wider implementation of PROs to maximize diversity of settings and patients and reduce potential health disparities in depression screening and care. In addition, our analyses by clinic setting should be considered in light of the high representation of patients seen in orthopedic clinics given that these clinics were the first to initiate depression screening. Second, differences in depression by race may be due to intersectionality with ethnicity, as a majority of “Other race” patients also identified as Hispanic (62.9%). Findings on ethnicity may not be generalizable to other regions of the United States, as Hispanics in the current study region are mostly of Puerto Rican origin (~70%). Puerto Ricans have been identified as having higher rates of depression compared to other Hispanic subgroups (29). Hispanics in the United States are more likely to be of Mexican origin (~60%) (30). Thus, future work should examine racial/ethnic disparities in depression screening and diagnosis in other regions. Third, we only examined depression screens at one point in time (i.e., the first depression screen completed by a patient), whereas some patients may have completed depression screens several times over the study period; this could mean that some patients were mis-classified in our study—specifically those we classified as false negative screens (i.e., negative screen, positive diagnosis). Fourth, we used the presence of a mood disorder diagnosis as a proxy for potential assessment and treatment of depression, which is a limited indicator and should be validated in future studies regarding its accuracy given the ease of assessing this variable in the EMR. Finally, only some of the patients in our sample completed PROs for physical and social function, thereby limiting our ability to examine benefits of universal screening with biopsychosocial PROs.

Our results indicate that universal screening for depression symptoms with PRO screens appears acceptable and has the potential to be clinically useful in detecting depression in diverse patient populations outside of behavioral health in a large medical center. However, other research has failed to document improved mental health outcomes in response to universal depression screening in primary care (31). Expanded delivery of PROs to include physical and social functioning as well as depression should be explored as to whether such a strategy produces beneficial outcomes; in particular, such an approach could be used to develop a clinically-relevant model for addressing patients' biological, psychological, and social needs in an integrated fashion across the health system. For example, while one patient may present for care in an orthopedic clinic, another in the Emergency Department, and other in primary care, a population-health approach that helps patients and clinicians address physical, psychological, and social health can promote person-centered and cost-effective care by sharing responsibility for these dimensions of health across the health system. Brief PRO screens, such as computerized adaptive tests (that are grounded in Item Response Theory) in the PROMIS self-report measures may be especially useful for this type of strategy because these measures each take <1 min to complete on average (low patient burden) but have strong psychometric properties and clinical norms to aid in interpretation. This type of health system strategy would be most effective if clinicians were prompted to communicate about biopsychosocial concerns and linkages could be made to connect patients with biopsychosocially-relevent resources in the health system and the community. Approaches might include behavioral interventions to promote improved patient-provider communication about life stressors and unmet social needs that can capitalize on digital health technologies, such as a tablet-based intervention that was shown to increase patient disclosure of unmet social needs in healthcare appointments (32, 33). Another approach could be “social prescribing” strategies, whereby medical providers “prescribe” social and wellness programs in the community, such as volunteering and social programs (34, 35). Given acceptability and utility of universal depression screening in detecting untreated depression, a next step could include a brief biopsychosocial PRO assessments delivered across settings in order to form the foundation of a person-centered, population-health management strategy for large health systems that will promote patient engagement in care, optimal healthcare utilization, and improved health.

## Data Availability Statement

The datasets presented in this article are not readily available because these data were produced as part of routine clinical care and cannot be shared outside our institution without a data use agreement. Requests to access the datasets should be directed to Kimberly A. Van Orden, kimberly_vanorden@urmc.rochester.edu.

## Ethics Statement

The studies involving human participants were reviewed and approved by University of Rochester, Research Subjects Review Board. Written informed consent for participation was not required for this study in accordance with the national legislation and the institutional requirements.

## Author Contributions

KV, KC, MH, and MW contributed to conception and design of the study. KF and AL organized the database. JL and CS performed the statistical analysis. KV and JL wrote the first draft of the manuscript. CS, KF, and MW wrote sections of the manuscript. All authors contributed to manuscript revision, read, and approved the submitted version.

## Funding

This project was supported by funding from the National Institute of Mental Health (T32MH020061, 1K23MH125078-01A1) and NCATS (KL2 TR001999). Funds for publication fees were provided the UR Medicine Quality Institute.

## Conflict of Interest

The authors declare that the research was conducted in the absence of any commercial or financial relationships that could be construed as a potential conflict of interest.

## Publisher's Note

All claims expressed in this article are solely those of the authors and do not necessarily represent those of their affiliated organizations, or those of the publisher, the editors and the reviewers. Any product that may be evaluated in this article, or claim that may be made by its manufacturer, is not guaranteed or endorsed by the publisher.
