# Supplementary material for: URMC Universal Depression Screening Initiative: Patient Reported Outcome Assessments to Promote a Person-Centered Biopsychosocial Population Health Management Strategy
Source: Front Psychiatry. 2022 Jan 11;12:796499. doi: 10.3389/fpsyt.2021.796499 (PMC8787088; doi:10.3389/fpsyt.2021.796499)
Supplement: Supplementary file 1 [file Table_1.DOCX]

Supplemental Table 1

*Demographics by clinic setting*

|  | Age groups | | Sex | | Race | | Ethnicity | |
| --- | --- | --- | --- | --- | --- | --- | --- | --- |
| Clinic Comparison | Older n(% within group) | χ^2^(df) | Female n(% within group) | χ^2^(df) | White n(% within group | χ^2^(df) | Hispanic n(% within group) | χ^2^(df) |
| Primary Care vs. Specialty |  | 2684.32 (1)*** |  | 4.46 (1)* |  | 5459.22 (1)*** |  | 643.33 (1)*** |
| Primary Care | 3,467 (12.8%) |  | 16,296 (60.1%) |  | 14,607 (55.0%) |  | 2,633 (10.3%) |  |
| Specialty | 8,267 (31.3%) |  | 15,625 (59.2%) |  | 21,806 (84.7%) |  | 1,085 (4.4%) |  |
|  |  |  |  |  |  |  |  |  |
| Orthopedics/Pain vs. Others^a^ |  | 40.27 (1)*** |  | 90.61 (1)*** |  | 6284.40 (1)*** |  | 1134.14 (1)*** |
| Ortho/Pain | 34,631 (25.6%) |  | 74,555 (55.2%) |  | 116,878 (87.3%) |  | 4,378 (3.3%) |  |
| Others | 15,409 (24.3%) |  | 36,426 (57.4%) |  | 45,138 (72.8%) |  | 4,036 (6.8%) |  |
|  |  |  |  |  |  |  |  |  |
| Oncology vs. Others^a^ |  | 1425.56 (1)*** |  | 967.08 (1)*** |  | 224.71 (1)*** |  | 40.98 (1)*** |
| Oncology | 1,803 (53.1%) |  | 1,006 (29.6%) |  | 3,104 (92.4%) |  | 66 (2.1%) |  |
| Others | 48,237 (24.7%) |  | 109,975 (56.3%) |  | 158,912 (82.5%) |  | 8,348 (4.5%) |  |

^a^ Others includes all other clinics besides the comparison.

*p < .05

***p < .001
